# *K*-shuff: A Novel Algorithm for Characterizing Structural and Compositional Diversity in Gene Libraries

**DOI:** 10.1371/journal.pone.0167634

**Published:** 2016-12-02

**Authors:** Kamlesh Jangid, Ming-Hung Kao, Aishwarya Lahamge, Mark A. Williams, Stephen L. Rathbun, William B. Whitman

**Affiliations:** 1 Department of Microbiology, University of Georgia, Athens, Georgia, United States of America; 2 Microbial Culture Collection, National Centre for Cell Science, Savitribai Phule Pune University, Pune, Maharashtra, India; 3 School of Mathematical & Statistical Sciences, Arizona State University, Tempe, Arizona, United States of America; 4 Institute of Biotechnology and Bioinformatics, Savitribai Phule Pune University, Pune, Maharashtra, India; 5 College of Agriculture and Life Sciences, Virginia Polytechnic and State University, Blacksburg, Virginia, United States of America; 6 Department of Epidemiology and Biostatistics, University of Georgia, Athens, Georgia, United States of America; Universidad Autonoma Metropolitana, MEXICO

## Abstract

*K*-shuff is a new algorithm for comparing the similarity of gene sequence libraries, providing measures of the structural and compositional diversity as well as the significance of the differences between these measures. Inspired by Ripley’s *K*-function for spatial point pattern analysis, the Intra *K*-function or IKF measures the structural diversity, including both the richness and overall similarity of the sequences, within a library. The Cross *K*-function or CKF measures the compositional diversity between gene libraries, reflecting both the number of OTUs shared as well as the overall similarity in OTUs. A Monte Carlo testing procedure then enables statistical evaluation of both the structural and compositional diversity between gene libraries. For 16S rRNA gene libraries from complex bacterial communities such as those found in seawater, salt marsh sediments, and soils, *K*-shuff yields reproducible estimates of structural and compositional diversity with libraries greater than 50 sequences. Similarly, for pyrosequencing libraries generated from a glacial retreat chronosequence and Illumina^®^ libraries generated from US homes, *K*-shuff required >300 and 100 sequences per sample, respectively. Power analyses demonstrated that *K*-shuff is sensitive to small differences in Sanger or Illumina^®^ libraries. This extra sensitivity of *K*-shuff enabled examination of compositional differences at much deeper taxonomic levels, such as within abundant OTUs. This is especially useful when comparing communities that are compositionally very similar but functionally different. *K*-shuff will therefore prove beneficial for conventional microbiome analysis as well as specific hypothesis testing.

## Introduction

The quantitative comparison of gene sequence libraries has become an essential component of hypothesis-driven ecological research. With the increased affordability of high-throughput sequencing, the analyses of the microbiomes of humans, soils and other ecosystems has focused less on descriptions of microbiomes and more on examining the underlying environmental and biological factors affecting their structures. With the introduction of LIBSHUFF [1] and other software packages, such as the analysis of molecular variance or AMOVA [2], ∫-LIBSHUFF [3], DOTUR [4], UniFrac [5], SONS [6], TreeClimber [7], LibraryCompare [8] and Metastats [9], statistical comparisons of communities can now be based on multiple approaches and algorithms. Generally, these comparisons are based on operational taxonomic units (OTUs), which are derived from the sequence datasets using two different approaches: alignment-dependent clustering based on distance cutoffs and an alignment-independent reference based on prior phylotype assignments [10]. While the former is computationally expensive, the latter suffers from the incompleteness of the reference databases, which at times can lead to a major portion of the microbiome left unassigned. Using mock sequence datasets represented in a two-dimensional space of circles and ellipses with known shapes and densities, a systematic evaluation of these tools showed that the current statistical toolbox has the ability to address specific ecological questions concerning the differences among microbial communities [11]. However, these algorithms posed some limitations for complex analyses. For instance, the methods that depend upon assignment of sequences to operational taxonomic units or OTUs require large samples to estimate richness. Similarly, methods that depend upon Monte Carlo testing procedures become computationally expensive when comparing large sequence libraries that are typical of high-throughput technologies. Lastly, there are also limitations in the sensitivity of the algorithms for detecting ecologically relevant differences.

To address these concerns, the utility of a new algorithm for comparison of gene libraries called *K*-shuff was assessed. *K*-shuff was designed and implemented in a Fortran program and is motivated by Ripley’s *K*-function [12], which is a powerful statistical tool for spatial point pattern analysis. It uses measures of distances among all pairs of sequences to reflect any aggregation of these sequences as would arise from samples comprised of closely related organisms. So, more information is retained when compared to LIBSHUFF which only uses the distance between each sequence and its nearest neighbor. In contrast to spatial point processes, the distances among gene sequences typically encompass hundreds of dimensions, where each sequence position comprises a separate dimension. Thus, some ideal properties, such as stationarity and isotropy, which are typically assumed in developing *K*-function theories, are not relevant. However, this limit does not hinder the usefulness of *K*-functions for this application. As indicated by Diggle et al. [13] and the results of our case studies detailed below, empirical *K*-functions still render meaningful scientific interpretations.

In this study, we perform a systematic evaluation of the reproducibility, sensitivity and power of *K*-shuff, using previously reported 16S rRNA gene sequence datasets obtained using the Sanger method and pyrosequencing and Illumina^®^ platforms. In addition, we compare its performance with LIBSHUFF, the first statistical tool for this purpose, as well as the most frequently used tool today, UniFrac. We show that when correctly employed, *K*-shuff allows testing of multiple hypotheses in a single process and has the potential to significantly improve our understanding of ecologically meaningful aspects of microbial communities.

## Materials and Methods

### The *K*-shuff Metric

*K*-shuff identifies spatial clustering based on the reduced second moment measure, or *K*-function [14, 15]. In general, the *K*-function is defined based on a distance measure, which in this context is set to be the evolutionary distance between the gene sequences. Denoting the evolutionary distance between sequences *i* and *j* by *d*_*ij*_, the empirical *K*-function for a library of sequences of size *N* is:

$$K\left(r\right)=\frac{1}{N\left(N-1\right)}\sum_{i=1}^{N}\sum_{j\neq i}^{N}I\left(d_{ij}\leq r\right),$$

where *I*(*E*) is an indicator function (= 1, when *E* is true; = 0, if otherwise), and *r* is any positive number on the real line of evolutionary distances. In essence, this *K*-function is the empirical cumulative distribution function of the evolutionary distances for a gene library. For every value of evolutionary distance *r* in the distance matrix, *K*(*r*) is the fraction of *d*_*ij*_ values less than or equal to *r*.

**Fig 1:**
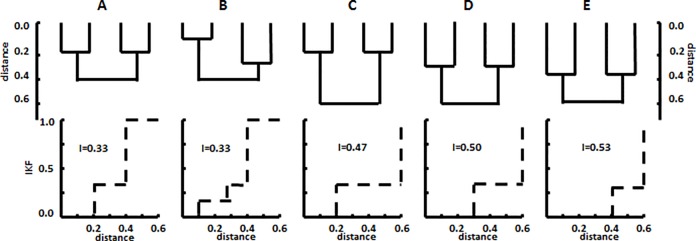
Intra *K*-functions or IKFs for hypothetical libraries of varying structural complexity. Each four member library is represented by its phylogenetic tree. Libraries A and B have the same overall diversity and the same *I*_kf_ value but different structures. For C, D, and E, increases in diversity correlate with increases in *I*_kf_.

The Intra *K*-function or IKF may be defined as above to describe the genetic diversity among the members of a single library, hereafter referred to its structure, and can be represented as a plot of *K*(*r*) as *r* increases from zero to its maximum value (Fig 1). For some applications, it is also convenient to describe the IKF as a single summary value, *I*_kf_, which represents the area above *K*(*r*) in the distribution plot (Fig 1). When a library has limited diversity, the IKF will rise rapidly and *I*_kf_ will be small, as shown in the comparison of Fig 1A and 1E. However, reducing the IKF to a single number necessarily causes loss of information, and multiple IKFs can yield the same *I*_kf_ value even when the libraries are significantly different, as seen in the comparison of Fig 1A and 1B. Moreover, in contrast to many conventional diversity estimates, the IKF is sensitive to both the number of OTUs and the relatedness between them. This explicit recognition of relatedness, not captured in binning methods, is an important advantage of the method’s ability to represent the community diversity, and a community comprised of many closely related sequences would have a lower *I*_kf_ than a community with same number of OTUs but comprised of distantly related sequences.

**Fig 2:**
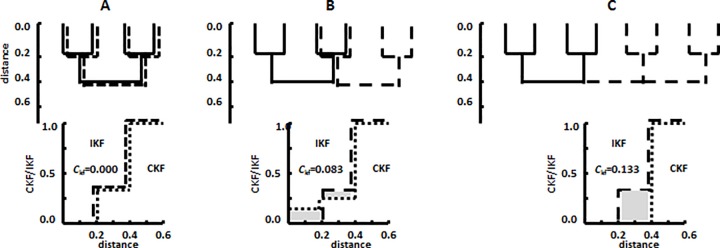
Cross *K*-functions or CKFs for hypothetical libraries of varying similarity. Each four member library is represented by its phylogenetic tree. All libraries have the same IKF, but the CKF can vary greatly. The *C*_kf_ is the sum of the area between each of the IKFs and the CKF or twice the shaded areas. A. Comparison of two identical libraries. B. Comparison of two libraries where half the members are the same. C. Comparison of two libraries where none of the members are the same. In this latter case, even though there is no overlap in the compositions of the two libraries, the members of the libraries possess limited diversity of 0.4.

While the IKF describes the genetic diversity or structure within each library, the Cross-*K*-function of CKF provides a natural measure of the dissimilarity in the membership or composition between paired libraries. In the CKF, the *K*-function is the computed distribution function of the evolutionary distances between pairs of sequences, one from each of the two libraries (Fig 2). When the compositions of the libraries are the same, it is possible to demonstrate that the expected value of the CKF is equal to the IKFs of the two libraries under random allocation or shuffling of members among the two libraries, i.e., the CKF is identical to the IKFs for each library. As the compositions of the libraries become more different, however, the CKF becomes more different from the IKFs. The magnitude of this difference can also be summarized by a single value *C*_kf_, which is the sum of the areas between each of the IKFs and the CKF (Fig 2).

### Theory behind *K*-shuff

Suppose that we have a collection of gene sequences and that the evolutionary distances *d*_*ij*_ have been computed for all pairs of sequences *i* and *j* in the collection. Separate empirical *K*-functions were designed to provide nonparametric summary measures of the richness or evenness of genetic diversity within a library and the dissimilarity of the composition between libraries. The first empirical *K*-function, the Intra *K*-function or IKF describes the genetic diversity of a library, *S*_*l*_. It may be regarded as the cumulative distribution function of evolutionary distances among members of the library *S*_*l*_. It is:

$$K^{\left(l\right)}\left(r\right)=\frac{1}{n_{l}\left(n_{l}-1\right)}\sum_{i=1}^{N}\sum_{j\neq i}^{N}I\left(d_{ij}\leq r\right)I\left(i\in S_{l}\right)I\left(j\in S_{l}\right)$$

Here, *n*_*l*_ is the number of sequences in the library *S*_*l*_ and *N* is the total number of sequences in all libraries being studied. *I*(*E*) is an indicator function that takes the value of 1 if *E* is true and 0 if otherwise. The quantity *d*_*ij*_ is the evolutionary distance between sequences *i* and *j*.

Plotting *K*^(*l*)^(*r*) against evolutionary distance *r* yields a unique description that more deeply summarizes the composition or diversity of the library relative to other diversity indices. For comparing large numbers of libraries, however, the summary diversity index

$$I_{kf}=1-\int_{0}^{\infty }K\left(r\right)dr,$$

may be computed, representing the area above *K*^(*l*)^(*r*) in the distribution plot. In libraries with low diversity, *K*^(*l*)^(*r*) will increase rapidly with increasing *r*, and *I*_*kf*_ will take a small value. In high-diversity libraries, *K*^(*l*)^(*r*) will increase slowly, and *I*_*kf*_ will take a large value.

For describing the dissimilarity in the membership between a pair of libraries $S_{l_{1}}$ and $S_{l_{2}}$, we consider the following bivariate empirical *K*-function, the Cross *K*-function or CKF. It is:

$$K^{\left(l_{1},l_{2}\right)}\left(r\right)=\frac{1}{n_{l_{1}}n_{l_{2}}}\sum_{i=1}^{N}\sum_{j=1}^{N}I\left(d_{ij}\leq r\right)I\left(i\in S_{l_{1}}\right)I\left(j\in S_{l_{2}}\right);$$

see also Diggle [16]. Here, $n_{l_{1}}$ and $n_{l_{2}}$ are the number of sequences in libraries $S_{l_{1}}$ and $S_{l_{2}}$, respectively. The quantity *d*_*ij*_ is now the distance between a sequence *i* of library $S_{l_{1}}$ and a sequence *j* of library $S_{l_{2}}$.

Plotting $K^{\left(l_{1},l_{2}\right)}\left(r\right)$ against evolutionary distance *r* yields a description of the compositional similarity of the two libraries. For pairs of libraries that are compositionally similar, $K^{\left(l_{1},l_{2}\right)}\left(r\right)$ will increase rapidly with increasing *r*. For dissimilar libraries, $K^{\left(l_{1},l_{2}\right)}\left(r\right)$ will increase slowly. This may be reduced to the index

$$C_{kf}=\int_{0}^{\infty }\left|K^{\left(l_{1},l_{2}\right)}\left(r\right)-K^{\left(l_{1}\right)}\left(r\right)\right|dr+\int_{0}^{\infty }\left|K^{\left(l_{1},l_{2}\right)}\left(r\right)-K^{\left(l_{2}\right)}\left(r\right)\right|dr,$$

which is the sum of the areas between each of the IKFs and the CKF.

To determine the homogeneity of the genetic diversity or structure among libraries *S*_1_,*S*_2_,⋯,*S*_*L*_, the following test statistic was used in analogy with an analysis of variance (ANOVA) test proposed by Cuevas et al. [17] for functional data:

$$T_{l}=\sum_{l=1}^{L}n_{l}{\int_{0}^{\infty }\left[K^{\left(l\right)}\left(r\right)-\bar{K}\left(r\right)\right]}^{2}dr$$

where $\bar{K}\left(r\right)=\frac{\sum_{l=1}^{L}n_{l}K^{\left(l\right)}\left(r\right)}{N}$ is the weighted average of the IKFs. When all libraries are sampled from the same population, the statistical expectations of all IKFs are the same as their average, resulting in a small *T*_*l*_. Otherwise, when the structures are very different, the *T*_*l*_-value will be large.

As for comparing the composition between libraries $S_{l_{1}}$ and $S_{l_{2}}$, we propose the following test statistic:

$$T_{c}^{\left(l_{1},l_{2}\right)}={\int_{0}^{\infty }\left[K^{\left(l_{1},l_{2}\right)}\left(r\right)-\frac{n_{l_{1}}K^{\left(l_{1}\right)}+n_{l_{2}}K^{\left(l_{2}\right)}}{n_{l_{1}}+n_{l_{2}}}\right]}^{2}dr.$$

This test statistic compares the CKF with the weighted average of the IKFs. We note that when the two libraries are sampled from the same community and thus possess similar compositions, their IKFs, $K^{\left(l_{1}\right)}(r)$ and $K^{\left(l_{2}\right)}(r)$, and the CKF, $K^{\left(l_{1},l_{2}\right)}(r)$ have identical expectations. Hence, the test statistic $T_{c}^{\left(l_{1},l_{2}\right)}$ is small. On the other hand, when the two libraries are sampled from very different communities, the difference between them as indicated by the CFK is much larger than the diversity within each library, represented by the IKF. The test statistic then becomes large.

Following Singleton et al. [1], a Monte Carlo procedure was used to approximate the distributions of *T*_*l*_ and *T*_*c*_ and obtain their corresponding p-values. Specifically, sequences are randomly shuffled across libraries for a large number of times, say 999 times. After each shuffling, *T*_*l*_ as well as *T*_*c*_-values are generated. The distribution of each test statistic is approximated from the frequency distribution of the (e.g., 999) generated *T*_*j*_-values. In addition, p-value can be approximated by the frequency of the generated *T*_*j*_-values that are greater than the observed one.

### Validation of *K*-shuff

#### Using *K*-shuff

*K*-shuff is available for download as Linux source code and Windows executable (current version 1.1) from https://research.franklin.uga.edu/whitman/content/k-shuff along with test datasets and the user manual. All analyses were performed on a personal computer with 2.8 GHz processor and 4 GB RAM. To run *K*-shuff, a PHYLIP-formatted distance matrix and a control file are required. Because *K*-shuff is capable of performing multiple comparisons in a single run, one should include all libraries in a single distance matrix. The control file is a simple text file containing parameter settings for the run, such as the path to the distance matrix file, number of libraries in the matrix, number of sequences in each library, etc.

#### 16S rRNA gene datasets

To demonstrate its general applicability and sensitivity, *K*-shuff was used to examine 16S rRNA gene libraries constructed using the Sanger method and pyrosequencing and Illumina^®^ platforms. These data sets were chosen to be representative of interesting bacterial communities that varied in composition as well as structural diversity. Libraries of 16S rRNA gene clone sequences for sea water and salt marsh sediments were from Lasher et al. [18]. These libraries comprised Sanger sequences of about 400 bp. Libraries for cropland, forest and grassland soils from Georgia, Kansas and Michigan were from Jangid et al. [19, 20, 21], respectively. Prepared by Sanger sequencing, the sequences were typically about 800 bp. Because all of these clone libraries were essentially prepared under an identical protocol, the influence of experimental variations on the observed results were expected to be minimal. For the high-throughput methods, microbial communities from soils formed by the retreating Franz Josef glacier on the South Island of New Zealand were sampled by pyrosequencing as described by Jangid et al. [22]. rRNA gene pyrosequences from this chronosequence had an average read length of 260 bp and are deposited in SRP006445.2. For the Illumina^®^ platform, sequences from the home life study by Dunn et al. [23] were used. First, from a total of 1,719,177 quality-filtered reads a random subset of 1000 reads per sample were extracted as used by the authors in their analysis, which resulted in 174,000 reads from 174 samples representing nine standardized locations within 18–20 different homes. For subsequent analyses of the effect of sample size, sequence subsets were then selected from the 1000 sequence data per sample. For the PCoA of *C*_kf_, 100 sequences were randomly subsampled from each library. Randomization from each of the 174 samples yielded between 95–100 sequences for a total of 17366 sequences, which were then used for the final analysis.

All sequences were aligned against the SILVA reference alignment within MOTHUR [24], and a PHYLIP-formatted squared distance matrix was prepared. Conventional diversity estimates were calculated using OTUs clustered at *D* = 0.03 using the average neighbor algorithm in MOTHUR.

#### General application of *K*-shuff

To test a common potential application of *K*-shuff, the composition of four relatively small libraries of bacterial 16S rRNA genes were compared. These 80 member libraries comprised sequences randomly selected from larger libraries constructed from estuarine seawater (SW) and salt marsh sediment (MS) from the Sapelo Island Microbial Observatory [18] and forest (FS) and cropland soil (CS) from the J. Phil Campbell Sr. Natural Resource Conservation Center in Georgia [20].

The differences between the communities were determined using non-metric multi-dimensional scaling (MDS) analysis in Statistica v10 (www.statsoft.org). For this, the values of the *C*_kf_ were used as an input matrix for the plots. In addition, PCoA of the compositional parameter (*C*_kf_) matrix using Sorenson distance was used to identify clustering of groups along ordination axes for the household microbiome (see below). A test that household bacterial communities were significantly different (α<0.01) from one another was determined using the multi-response permutation procedure (MRPP) in PC-ORD Version 6 [25]. A measure of the effect size, based on a chance corrected within group-agreement (A-value) was used to assess heterogeneity within and between groups.

#### Power analysis and simulations of *K*-shuff performance

To evaluate the utility of *K*-shuff more systematically, larger 16S rRNA gene libraries were compiled from both Sanger and high-throughput datasets. Clone library simulations were carried out by combining 1000 sequences from each of SW, MS and soils (FS+CS) that were representative of communities that varied in composition as well as structural diversity (Fig A in S1 File). Pyrosequences were obtained from a soil chronosequence dating from 60 y to 120 ky at the Franz Josef glacier on New Zealand [22]. For each of the nine ages in the chronosequence, libraries of 2000 sequences were prepared from each of five replicate samples or 10000 for each age. To evaluate the number of sequences required for *K*-shuff, 100, 200 or 300 sequences were randomly selected from each replicate, and the sequences from replicates were pooled for *K*-shuff analyses of 500, 1000, and 1500 sequences for each age.

Similar simulations were also carried out on the Illumina^®^ datasets. Thus, the least number of sequences required for a reproducible measure of IKF and CKF was determined from random samples of 100, 200, 300, 400 and 500 sequences per sample for Cutting board (19 samples), Pillowcase (20 samples), and Door Exterior (18 samples) communities for a total of 57 samples. These sites were chosen because they varied in diversity and showed low, intermediate, and high OTU richness, respectively (see Fig 1 in Dunn et al. [23]).

Power analysis was then performed to test the statistical power of *K*-shuff by randomly selecting sequences from the libraries in different proportions (ω) to create pairs of libraries, where the total number of sequences in each library is N. For instance, for comparison of the SW and MS libraries, one library in each pair consisted of ωN SW and (1-ω)N MS sequences, while the other library in the pair contained (1-ω)N SW sequences and ωN MS sequences. With ω = 0.5, the two generated libraries should be similar in both structure and composition because both contained random selections of the same number of sequences from each environment. Then ω was increased from 0.5 to 1.0 in steps of 0.05, resulting in pairs of libraries with increasing levels of dissimilarity. When ω = 0.70, the SW library of N = 100 contained 70 sequences from SW and 30 sequences from MS. The paired MS library contained 70 sequences from MS and 30 sequences from SW. Thus, both libraries contained 30 SW and 30 MS sequences. When ω = 1.0, the libraries had their maximum difference because there was no mixing. For each combination of N and ω, the *T*_*I*_ and *T*_*c*_ test statistics for the *I*_kf_ and *C*_kf_, respectively, were calculated on 1000 pairs of mixed libraries, and the fraction of comparisons that were significantly different or the power was calculated.

## Results

### General application of *K*-shuff

**Fig 3:**
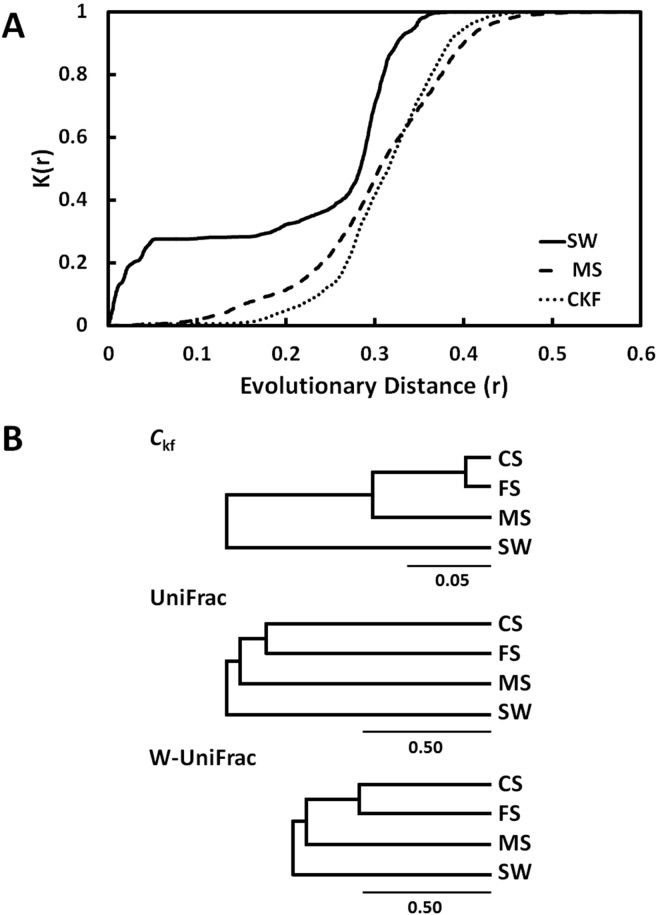
Comparing 16S rRNA gene clone libraries using *K*-shuff. A. Measurement of structural and compositional differences between the 80-member libraries from seawater and marsh sediment bacterial communities using the intra *K*-functions or IKFs (labeled SW or MS) and the cross-*K*-function (or CKF). A CKF which is ‘flat’ at low evolutionary distances indicates a large dissimilarity in the composition between the two corresponding libraries. B. UPGMA trees of the *C*_kf_ and unweighted (UniFrac) and weighted UniFrac (W-UniFrac) distances for the bacterial communities.

*K*-shuff was first evaluated using 80-member clone libraries of Sanger sequences generated from seawater (SW), marsh sediment (MS), cropped soil (CS) and forest soil (FS) bacterial communities. Because of their longer reads, Sanger libraries better represent the taxonomic diversity of bacterial communities than many NexGen libraries [26]. These libraries were chosen to represent both simple and complex communities and communities from different environments. The SW community was composed of groups of closely related OTUs drawn from just a few phyla and had a relatively low diversity (Fig 3A). This was reflected in the IKF by the rapid increase in K(r) at low levels of evolutionary distance, followed by a plateau and then increase at high levels of evolutionary distance of about 0.3. In contrast, the diversity of the MS community was much greater, and the IKF remained low at low levels of evolutionary distance. The IKFs for the soil communities were very similar to those of the MS. These general observations were also reflected in the *I*_kf_ values, which were 0.214, 0.302, 0.290, and 0.283 for the SW, MS, CS, and FS communities, respectively. While the differences between the SW *I*_kf_ and the other values were all significant at p <0.001, the *I*_kf_ of the two soil communities were not significantly different from each other (p = 0.820) or that of the MS (p = 0.18 and 0.09, respectively). These analyses confirm those of Lasher et al. [18], who had shown that the structural diversity of the bacterial communities of MS from Sapelo Island and soils were similar.

Although the structures of the MS and soil communities were not significantly different, their compositions differed significantly from each other as well as the SW community with p≤0.003. For the comparison of the SW and MS communities, this difference was reflected in the CKF, whose value remained low at low evolutionary distances or *r* values before rapidly increasing at high levels of *r* (Fig 3A). Although the compositions of all the communities were significantly different, the magnitude of the differences varied greatly. For instance, the *C*_kf_ value between the soil communities was 0.020, compared to 0.06–0.09 and >0.16 for comparisons between the soil and MS and SW communities, respectively (Fig 3B). Consistent with the low *C*_kf_ value, the soil communities possessed many OTUs in common and had a similar overall composition of their major phylogenetic groups [20]. UniFrac also indicated a similar hierarchical clustering of these communities (Fig 3B). However, the p-values were only marginally significant (p≤0.06). Moreover, the magnitude of the difference between the soil communities was nearly equal to their differences from the MS community for either the unweighted or weighted UniFrac distances, which was not consistent with comparisons of either the major phylogenetic groups or OTUs present [18, 20]. Thus, *K*-shuff appeared to provide a better representation of the compositional diversity among these communities.

### Power analysis and simulations of *K*-shuff performance with clone libraries

**Fig 4:**
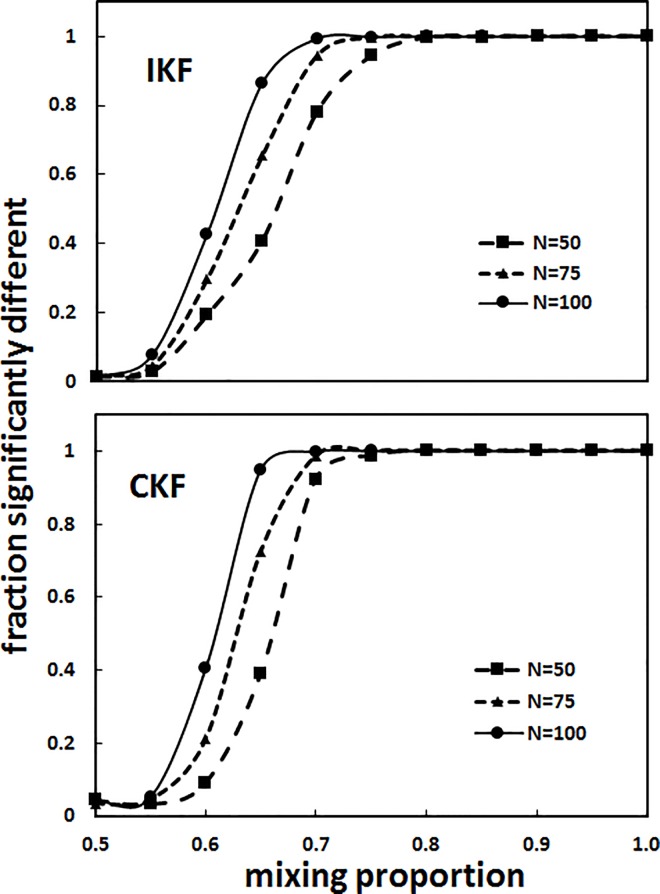
Power of *T*_*I*_ (IKF) and *T*_*c*_ (CKF) to distinguish libraries of bacterial 16S rRNA gene sequences from seawater (SW) and salt marsh sediment (MS) communities. The fraction of 1000 *K*-shuff comparisons that were significantly different (p ≤ 0.05) or the power compared to the mixing proportion (ω) of the libraries. N = number of sequences in each library.

To determine the sensitivity of K-shuff to differences in the composition of the libraries, power analyses were performed by mixing the libraries from different communities. The expectation was that a sensitive statistic would detect differences in the libraries at a low mixing ratio. The power curves for both *T*_*I*_ and *T*_*c*_, the test statistics for the *I*_kf_ and *C*_kf_ respectively, increased rapidly with the mixing proportion (or ω) for the MS and SW libraries, indicating that *K*-shuff readily distinguished libraries whose structures and compositions differed (Fig 4). For instance, at a mixing proportion of 0.70, the power of *T*_*I*_ to distinguish structural differences between the libraries was already 0.782 for N = 50, 0.945 for N = 75, and 0.994 for N = 100, where N is the size of the libraries (Fig 4 and Table A in S1 File). Similarly, the power of *T*_*c*_ to distinguish compositional differences was above 0.90 even at N = 50. Compositional differences between the MS and soil bacterial communities were also readily detected. At a mixing proportion of 0.70, the power was 0.698 for N = 50, 0.901 for N = 75, and 0.998 for N = 100 (Table B in S1 File). Previously, the sediment libraries had been found to possess a structural diversity comparable to soil [18]. Consistent with this result, the power of *T*_*I*_ remained low under all the conditions tested (Table B in S1 File). In conclusion, the test statistics had a high power to detect small differences in the libraries, and compositional differences were detected in the absence of large structural differences.

*K*-shuff was also more sensitive to small changes in the library composition than widely used alternative methods. Based upon 50 replicate libraries, the power of *K*-shuff to detect significant differences between SW and MS libraries with ω = 0.70 was 0.92, or essentially the same as with 1000 replicates. In contrast, the powers of LIBSHUFF and UniFrac under the same conditions were 0.32 and 0.16, respectively. Thus, *K*-shuff was much more sensitive that either of these two commonly used methods.

It can be demonstrated that the expected values of both *C*_kf_ and *I*_kf_ do not depend on sample size. However, variation in the means is expected as a consequence of higher sampling variances in the smaller samples. Simulations were performed to determine the effect of the sample size N on the sampling variation in *I*_kf_ and *C*_kf_. Sampling variation in *K*-shuff decreased with increasing N. For instance, the mean ± standard errors of *C*_kf_ for comparisons of soil and MS were 0.065 ± 0.015, 0.064 ± 0.010, 0.063 ± 0.008, and 0.063 ± 0.007 for N of 25, 50, 75, and 100, respectively (Fig B in S1 File). This effect was also observed for the *I*_kf_, of the soil and marine sediments libraries (Figs C and D in S1 File). For instance, the mean ± standard error of the *I*_kf_ for soil libraries were 0.268 ± 0.019, 0.275 ± 0.012, 0.277 ± 0.009, and 0.277 ± 0.008 for N of 25, 50, 75, and 100, respectively. An important implication of this result is that the libraries do not have to be the same size to compare these parameters.

If the *C*_kf_ measured the difference between libraries, it would be expected to increase as the proportion of mixing or ω increased from 0.5 to 1.0 and the libraries became more different. However, the relationship between *C*_kf_ and mixing should be complex, reflecting also how the libraries’ structures affected the calculation of *C*_kf_. At proportions of 0.5, the *C*_kf_ was close to twice the standard error, reflecting the fact the *C*_kf_ is always a positive number or zero and the means for measurements of random libraries must always be greater than zero (Fig E in S1 File). However, at high values of ω, a strong relationship was seen between ω and the *C*_kf_, as expected if *C*_kf_ were a reliable indicator of differences between libraries.

If the *I*_kf_ reflected the structures of libraries, it would also be expected to become more like the highly represented library as the mixing proportion increased. Like the *C*_kf_, the relationship between *I*_kf_ and ω was expected to be complex. First, the relationship between *I*_kf_ and mixing proportion of the libraries was examined in the seawater and sediment libraries, which were very different. As the mixing proportion increased from 0.5 to 1.0, the *I*_kf_ of the “seawater” library decreased from 0.227 to 0.181, and the *I*_kf_ of the “sediment” library increased from 0.227 to 0.305, and there was a clear trend between *I*_kf_ and ω (Fig F in S1 File). In contrast, as dramatic a trend was not found for the soil and sediment libraries, whose structures were very similar. When the proportion of mixing was 0.5, the *I*_kf_ was very close to that of the sediment, and the “sediment” library remained close to this value as its proportion increased. The *I*_kf_ of the “soil’ library decreased from 0.304 to 0.277 as its proportion increased to 1.0. Thus, while the expected trends were observed, the changes in *I*_kf_ were not close to linear with the proportion of each library.

### *K*-shuff performance with Illumina^®^ dataset

Similar to the Sanger sequences from clone libraries, only small numbers of sequences were needed to calculate the *K*-shuff functions. For instance, the *I*_kf_ values calculated for random samples of 100–500 sequences from the cutting board, door trim and pillowcase were very similar (Fig G in S1 File). Additional simulations were then performed using the cutting board (Cb) and pillowcase (Pc) libraries, which appeared to be typical of the diversity within the datasets and a good test of the sensitivity of *K*-shuff towards structural and composition differences [23]. Similar to the clone libraries, simulations on the reproducibility and effect of sample size on the *I*_kf_ and *C*_kf_ found that the mean values were relatively constant but the sampling variation decreased as N increased from 50 to 100. For instance, the mean ± standard deviation of *C*_kf_ for comparisons between the cutting board (Cb) and pillow case (Pc) libraries were 0.024 ± 0.008, 0.024 ± 0.006 and 0.024 ± 0.005 for N of 50, 75 and 100, respectively (Fig H in S1 File). The small *C*_kf_ values emphasized that the compositions of these libraries were very similar. Likewise, the mean *I*_kf_, ± standard deviation of the Cb and Pc libraries were 0.198 ± 0.006, 0.198 ± 0.005 and 0.198 ± 0.004 and 0.220 ± 0.006, 0.220 ± 0.005 and 0.220 ± 0.005 for N 50, 75, and 100, respectively (Figs I and J in S1 File).

Power analysis for the Illumina^®^ dataset was performed using the cutting board (Cb) and pillowcase (Pc) libraries. While the fraction of significantly different *I*_kf_ and *C*_kf_ values increased with the mixing proportions, the increases were gradual, consistent with the small differences between the samples. For instance, structural differences between the libraries were only consistently observed at high mixing proportions (Fig K and Table C in S1 File). Similarly, compositional differences between the libraries were only consistently observed for the largest libraries of N = 100 (Fig K in S1 File). These results suggested that it was still possible to detect small differences in *I*_kf_ and *C*_kf_ at high mixing proportions even with small sample sizes. Similar to the trends for clone library comparisons, the changes in *C*_kf_ and *I*_kf_ were correlated with the mixing proportion of each library (Figs L and M in S1 File).

### *K*-shuff performance with pyrosequencing dataset

For this data, much higher numbers of sequences were required to obtain consistent *C*_kf_ values. While there was a good correlation, with a *r*^*2*^ of 0.92, between the *C*_kf_ values for the 1000 and 1500 sequence libraries, the correlations for the 500 sequence libraries were much lower, *r*^*2*^ = 0.44 and 0.50, with the 1000 and 1500 sequence libraries, respectively. This result suggested that 500 sequences were not sufficient for reproducible estimates of *C*_kf_ values with the pyrosequencing libraries. Thus, the libraries composed of 1500 sequences were used for additional analyses (see below). Because fewer sequences were required for reproducible estimates of *C*_kf_ values for both Sanger libraries and Illumina sequencing, the requirement of large numbers of sequences seems to inherent to either pyrosequencing per se or the associated methods of sample preparation.

### Application of *K*-shuff to studies of soil bacterial communities

A total of 126 16S rRNA gene libraries prepared by Sanger sequencing from soils collected in Georgia, Kansas and Michigan were analyzed by *K*-shuff [19, 20, 21]. Each of the three sites was represented by seven treatments, which included different types of vegetation, land management, and soil. For each treatment, six libraries were constructed, three from replicate plots sampled in the summer and three from the same plots sampled the following winter. Each sample comprised five cores, which were pooled prior to DNA extraction and library construction. ANOVA of the *C*_kf_ confirmed previous observations that the treatments had significant effects on the compositions of the bacterial communities [19, 20, 21]. However, the magnitude of the treatment effects depended on the site, with sites with the largest differences in land use having the largest effects (Table 1). Thus, treatment effects in Georgia, where the sites included cropland, pasture and forest, were larger than those in Kansas, where the treatments corresponded to just cropland and prairie. In contrast, the effect of season all three sites was modest. Notably, the variation among the replicates was only somewhat lower than the variation between treatments even though only 56 out of the 126 CKFs between replicates were significantly different at p<0.05 (Table 1). This result suggested that ‘noise’ limited the ability to observe small differences in these communities. Because technical replicates were not performed, it was not possible to determine if the noise was due to variation in the PCR, cloning, and other aspects of the library preparation and sequencing or the inherent variability of the bacterial communities.

**Table 1:** ANOVA analyses of *C*_kf_ values for 126 soil libraries prepared by Sanger sequencing.

	Georgia (Mean ± SE)	Kansas (Mean ± SE)	Michigan (Mean ± SE)	All^a^ (Mean ± SE)
**Treatment**	0.0199 ± 0.0019	0.0086 ± 0.0012	0.0128 ± 0.0019	0.0138 ± 0.0010
**Season**	0.0074 ± 0.0033	0.0070 ± 0.0021	0.0066 ± 0.0033	0.0070 ± 0.0018
**Replicates**	0.0157 ± 0.0013	0.0132 ± 0.0008	0.0172 ± 0.0013	0.0154 ± 0.0007
**p-value**	0.00600	0.00257	0.00904	0.00015

^a^ For the ANOVA of the combined samples or ‘All’, the N for treatment, season and replicates were 21, 42 and 126, respectively.

**Fig 5:**
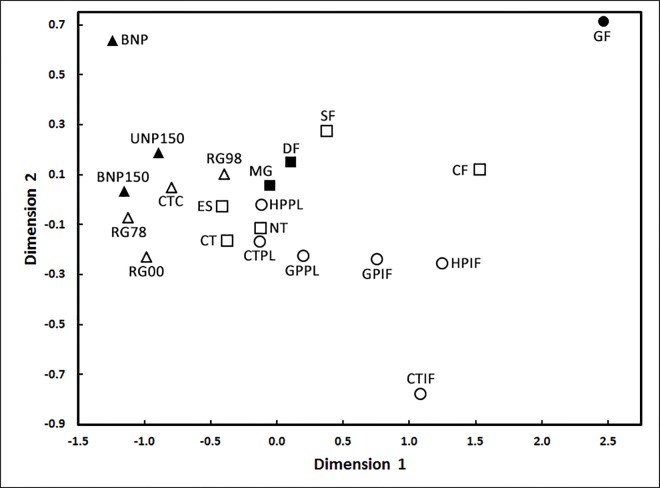
MDS plot of the compositional parameter *C*_kf_ for soil bacterial communities from Georgia (circles), Kansas (triangles), and Michigan (squares). Open and closed symbols are for disturbed and undisturbed soils, respectively. For treatment designations, see main text. For treatment details, see Jangid et al. [19, 20, 21].

Although *K*-shuff indicated that, with one exception, the composition of the bacterial communities from all the treatments at all three sites were significantly different, for many communities differences were small. Only the comparison between the Kansas BNP (burned native prairie [300 m^2^ plot size]) to UNP150 (unburned native prairie [150 m^2^ plot size]) was not significantly different (p = 0.054), while all other comparison had p-values below 0.01. However, in the MDS plot of the *C*_kf_ values (Fig 5), a large cluster of communities of similar composition were found in soils from Georgia (cropland and pastures amended with poultry litter [CTPL, HPPL, and GPPL]), Michigan (conventionally and no-tilled cropland [CT and NT], cropland allowed to become fallow or early successional [ES], native meadow [MG], successional forest (SF), and deciduous forest [DF]), and Kansas (conventional cropland [CTC], and prairie restored in 1978 [RG78], 1998 [RG98], and 2000 [RG00]). This cluster of similar communities was well separated from those in the native prairie (BNP) in Kansas, cropland and pastures amended with inorganic fertilizer (CTIF, HPIF, and GPIF) in Georgia, forest which had not been disturbed other than for logging for over 135 years (GF) from Georgia, and a coniferous forest plantation (CF) in Michigan. Surprisingly, little similarity was found in many of the bacterial communities from soils with similar management or vegetation, such as the forest communities in Michigan (DF) versus Georgia (GF) or the conventional croplands in Michigan (CT) and Kansas (CTC) versus Georgia (CTIF).

A variety of diversity indices are commonly used for comparisons of the structural or genetic diversity in gene libraries. While agreement of these indices is seldom perfect, they frequently give similar estimates. The *I*_kf_ estimates also correlated with other diversity indices, as shown for the J. Phil Campbell Sr. Natural Resource Conservation Center site in Georgia (Table 2). For instance, all the indices tested agreed that the forest and one of the croplands (CTIF) had the lowest diversity. However, only the *I*_kf_ consistently indicated that soils amended with poultry litter had a higher diversity than soils amended with inorganic fertilizer. This conclusion was supported by analysis of the compositional diversity, which found that a number of bacterial groups were more abundant in the litter-amended soils [20]. Moreover, the absence of complete agreement between the indices was not surprising. The *I*_kf_ was the only index that also considered the relatedness of the OTUs in addition to their number.

**Table 2:** Using the structural parameter *I*_kf_ with conventional diversity indices for soil bacterial communities^a^.

Diversity Index	Cropland		Hayed Pasture		Grazed Pasture		Forest
	CTIF	CTPL	HPIF	HPPL	GPIF	GPPL	GF
**N**^**b**^	259	263	272	275	274	260	277
**S**^**c**^	107	195	195	197	184	200	142
***I***_**kf**_^**d**^	0.279^x^	0.295 ^y^	0.281 ^x^	0.297 ^y^	0.277 ^x^	0.301 ^y^	0.260 ^z^
**H**^**e**^	4.37	5.04	5.11	5.13	4.98	5.14	4.56
**1/D**^**f**^	71	125	265	262	150	267	77
**Chao1**^**g**^	125	752	911	529	534	1040	382
**95% lci**^**h**^	115	517	585	397	389	662	269
**95% hci**^**i**^	145	1068	1351	708	733	1547	546

^a^ Soils were from the J. Phil Campbell Sr. Natural Resource Conservation Center in Watkinsville, Georgia [20]. CTIF, HPIF and GPIF were inorganic fertilizer-amended soils and CTPL, HPPL and GPPL were poultry litter-amended soils. GF was a nearby forest soil that had not been tilled since 1860. Only libraries from triplicate soil cores collected in summer were combined for these analyses.

^b^ N = number of clones in the library.

^c^ S = number of OTUs formed at *D* = 0.03 using DOTUR [4].

^d^ Values with different superscripts were significantly different, p = 0.05

^e^ Shannon diversity index, H = Σ[(n/N)ln(n/N)].

^f^ Simpson’s index, D = Σn(n-1)/N(N-1)

^g^ Chao 1 = S + n_1_^2^/2n_2_, where n_2_ is the number of clones that occur twice.

^h^ 95% lower confidence interval for Chao 1 estimator.

^i^ 95% higher confidence interval for Chao 1 estimator.

**Fig 6:**
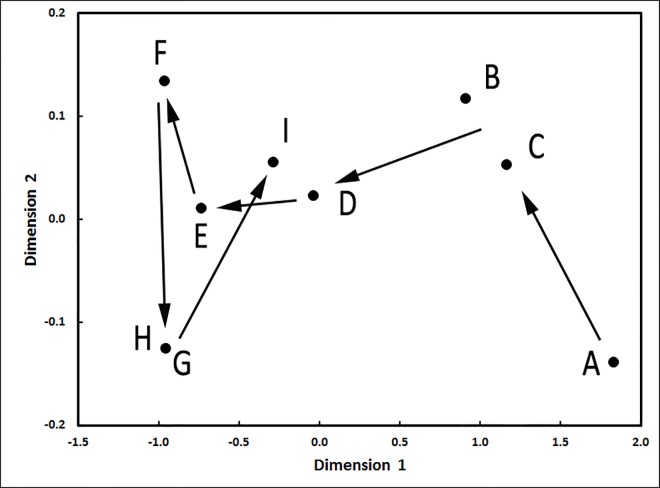
MDS plot of the compositional parameter *C*_kf_ for pyrosequencing libraries of the soil bacterial communities from the Franz Josef glacial chronosequence. The ages of the soils are: A, 60 y; B, 130 y; C, 280 y; D, 530 y; E, 1k y; F, 5k y; G, 12k y; H, 60k y; and I, 120k y. At each time point, five replicate soil samples were extracted and five libraries were constructed [22]. For this analysis, 300 sequences were randomly selected from each replicate and pooled, yielding 1500 sequences for each age in the chronosequence.

To determine if *K*-shuff would provide more insight into pyrosequencing datasets, libraries from a soil chronosequence dating from 60 y to 120 ky at the Franz Josef glacier on New Zealand were analyzed [22]. Previously, Bray-Curtis analysis of the 250 most abundant OTUs suggested that the soil communities were largely described by a monotonic function of change with age and failed to detect significant differences between the soil ages of 130 and 280 years; 530, 1k, and 12k years; and 5k, 12k, 60k, and 120k years [22]. In contrast, the CKF comparisons between the soil ages were all significantly different with p≤0.001, and the *C*_kf_ values varied within the range of 0.0028 and 0.0317. The MDS plot of the *C*_kf_ values demonstrated complicated changes in the composition of the soil bacterial community with increasing age across the chronosequence (Fig 6). Thus, *K*-shuff revealed much more detail about the compositional differences between the bacterial communities. Previous analyses also noted a progressive decline in the structural diversity during the first thousand years [22]. Similar changes were also seen in the *I*_kf_, which declined from 0.204 to 0.176 in the first thousand years and remained relatively constant after that.

### Application of *K*-shuff to studies of the home microbiome

Reanalysis of the home microbiome dataset of Dunn et al. [23] was performed to determine if *K*-shuff could recapture the major conclusions and provide new insights. Using 100 sequences per sample for all 174 samples, the *I*_kf_ were calculated for comparison to the OTU richness estimated by Dunn et al. [23]. Within each site, the OTU richness varied 3-10-fold, and the mean OTU richness of different sites varied nearly four-fold. In contrast, the *I*_kf_ values within each site varied less than 3-fold, and the mean *I*_kf_s were relatively constant, varying only from 0.15–0.21 (Fig N in S1 File). The differences in OTU richness and *I*_kf_s would be expected if much of the variation was due the presence or absence of rare OTUs, which have little effect on the *I*_kf_. Moreover, *I*_kf_s of about 0.20 resembled those of seawater, which is consistent with the low diversity expected of the microbiome of surfaces. Thus, the *K*-shuff analyses suggested that the diversity of the various household surfaces were fairly similar, which was not readily apparent from examination of only OTU richness.

**Fig 7:**
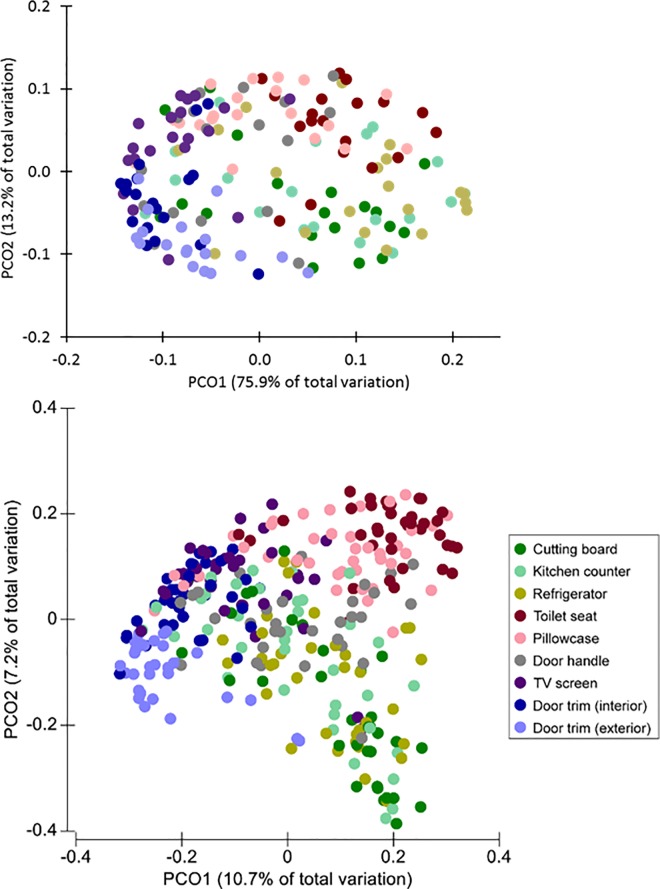
PCoA plots of the household bacterial communities with the compositional parameter *C*_kf_ for the subsampled Illumina^®^ libraries (top) and the unweighted UniFrac distances (bottom). For *C*_kf_ analysis, 100 sequences were randomly selected from each site per household and analyzed. For UniFrac, the complete data set by Dunn et al. [23] was used.

Similarly, PCoA plot of the variation among sites within the homes using the *C*_kf_ of *K*-shuff was compared to the original analysis using the UniFrac distances by Dunn et al. [23] (Fig 7). The MRPP of the compositional parameter (*C*_kf_) matrix from the household bacterial communities was significantly different (α<0.01), with clusters represented by PCoA. Each of the bacterial communities from different household habitats were patterned along two axes explaining 75.9 (Axis 1) and 13.2% (Axis 2) of the variance. A measure of the effect size based on a chance corrected within group-agreement test (A = 0.2) indicated that heterogeneity between groups was greater than that within groups. These results are independent of sample size and thus help to support the veracity of the p-values. Likewise, the MDS plot of the *C*_kf_ showed similar clustering as the PCoA (Fig O in S1 File). Overall, these results indicated that ordination using the *C*_kf_ matrix explained a much greater proportion of the variance than did analysis using UniFrac.

**Fig 8:**
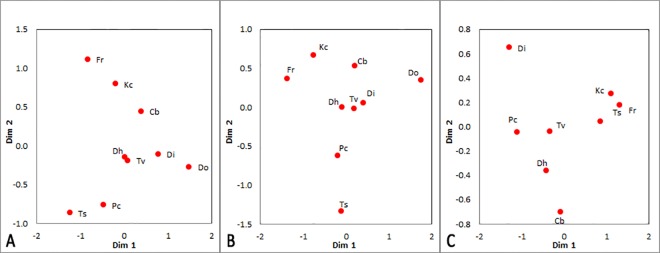
MDS plots of the compositional parameter *C*_kf_ for the subsampled Illumina^®^ libraries of the household bacterial communities. (A) for the entire sampled dataset, N = 17366; (B) for OTU1_0.14_ (the largest OTU at *D* = 0.14), N = 4271; and (C) OTU1_0.03_ (the largest OTU within OTU1_0.14_ at *D* = 0.03), N = 896. Do was removed from (C) because it had a single sequence. The nine sites are: Cb = Cutting Board; Kc = Kitchen Counter; Fr = Refrigerator; Ts = Toilet Seat; Pc = Pillowcase; Dh = Door Handle; Tv = Television Screen; Di = Door Interior; Do = Door Outer.

To utilize the extra sensitivity afforded by *K*-shuff, analyses were performed on some of the abundant OTUs to explore differences within the members of an OTU across the nine sites. At D = 0.14, a total of 4664 OTUs were found for the entire subsampled dataset (N = 17366), with the largest OTU (OTU1_0.14_) comprising 4271 sequences affiliated with the *Firmicutes*. Based on MDS analysis of the *C*_kf_s, OTU1_0.14_ showed a similar clustering pattern as the entire subsampled dataset (Fig 8A and 8B). Communities from sites Dh, Di and Tv were tightly clustered whereas Fr, Kc, Cb and Ts, Pc were spread across the plot in two loose clusters. It is worth noting that even though only six *C*_kf_ s values were >0.01 (range 0.0033–0.0848), p-values for all comparisons except those between Di and Tv (*C*_kf_ = 0.0033) were significant (p≤0.005). This result indicated that the composition of OTU1_0.14_ varied between many of the sites. The implication is that differences between the sites included differences in both the abundance and composition of this OTU.

Within OTU1_0.14_ there were 126 OTUs at D = 0.03 with 897 sequences in the largest OTU, namely OTU1_0.03_. MDS analysis of OTU1_0.03_ was very different from that of OTU1_0.14_ (Fig 8C). As expected, most of the *C*_kf_s calculated for comparisons across the sites were small (range 0.0005–0.0085) except for eight comparisons involving the outer door trim (Do). However, only one OTU1_0.03_ sequence was found at Do, and none of the *C*_kf_ values involving it were significantly different (Table D in S1 File). For that reason, Do was not included in the MDS plot. Nevertheless, these results indicated significant variation in the composition of even this very small group between the samples. For instance, the composition of OTU1_0.03_ on the interior door trim (Di) was significantly different from that of the kitchen counter (Kc), refrigerator (Fr) and toilet seat (Ts).

## Discussion

### Comparison of *K*-shuff to other methods

*K*-shuff is a sensitive method for detecting small but significant differences in the structural and compositional diversity in gene libraries. Like LIBSHUFF, UniFrac, P-test, F_ST_ and TreeClimber, it tests the hypothesis that two communities are alike [1, 2, 3, 5, 7, 27]. All the methods begin with similarity matrices of sequences in gene libraries. However, UniFrac, P-test and TreeClimber generate phylogenetic trees, whose properties are then tested [11]. We specifically compared *K*-shuff with LIBSHUFF because both methods test the similarity matrices directly, and with UniFrac because it is currently one of the most frequently used tool. The *K*-shuff IKF and CKF are analogous to the homologous and heterologous coverages as defined in LIBSHUFF [1]. However, while LIBSHUFF only utilized the evolutionary distances between each sequence and its closest neighbor, the *K*-functions use information on evolutionary distances among all members of the libraries. Therefore, the *K*-function uses more of the information contained in the libraries, which may account for its higher sensitivity. Moreover, unlike the heterologous coverage used in LIBSHUFF, the *C*_kf_ is symmetric or independent of the direction of the comparison, i.e. $K^{\left(l_{1},l_{2}\right)}(r)=K^{\left(l_{2},l_{1}\right)}(r)$. Symmetry is a sensible property since both *C*_kf_s provide the same information, i.e., the difference in the membership between the two libraries, and should agree with each other. However, unlike UniFrac which detects any differences that tend to assign the total branch length of a tree to a particular community [Schloss 2008], the sensitivity of *K*-shuff allows for an in-depth analysis of taxa that contribute to differences between communities. In our studies, UniFrac proved even less sensitive than LIBSHUFF, presumably because the process of generating phylogenetic trees greatly reduces the information content of the similarity matrices. Thus, *K*-shuff gives a more realistic assessment of differences in the communities.

Because of its high sensitivity, *K*-shuff is especially informative with relatively small libraries, such as those generated by Sanger sequencing. However, even for NexGen libraries, which typically include thousands or millions of sequences, *K*-shuff may provide valuable insights. Its high sensitivity makes it possible to analyze small subsets of large datasets that might be particularly interesting, such as moderately abundant OTUs. It would also be suitable for examining large numbers of samples generated by multiplexing, which also yield small numbers of sequences for each library [28, 29, 30]. Because sample size has only a small effect on either *I*_kf_ or *C*_kf_ values, it is also practical to test subsamples of very large libraries, which is computationally much less expensive.

Similar to the most frequently used phylogenetic metric UniFrac [5], *K*-shuff also produces an estimate of distance between the communities that is suitable for ANOVA and clustering analyses. However, *C*_kf_, the distance estimate of *K*-shuff, gives equal weight to both closely related as well as distantly related organisms, thereby allowing a unbiased estimation of the community composition. In contrast, UniFrac gives higher weight to differences arising from distantly related organisms [31]. Another concern with UniFrac is that the distance estimates are dramatically nonlinear. For instance, in mixing simulations of seawater and salt marsh sediment libraries, the UniFrac distances were 0.08, 0.81 and 0.86 at mixing proportions of 0.5, 0.7 and 1, respectively, and were not closely related to the differences in composition of the libraries. In contrast, the *C*_kf_ values of *K*-shuff were 0.015, 0.049 and 0.121 at the same mixing proportions. Thus, *K*-shuff is more reflective of the differences in community composition.

### *I*_kf_ as a novel estimate of structural diversity

*K*-shuff provides *I*_kf_, which is a novel estimate of the structural diversity that reflects both the relatedness among OTUs or phylogenetic diversity as well as their relative abundances. Thus, a community comprised of many closely related sequences would have a smaller *I*_kf_ than a community with same numbers of OTUs but comprised of distantly related sequences. Essentially this would not lead to change in memberships for an OTU thereby maintaining both the evenness and richness of the two communities. Further, because many commonly used indices, such as Shannon, Simpson, and Chao 1 are not a measure of phylogenetic diversity, their values would only change with changing richness, evenness or a larger sample size [32]. In addition, these estimates are biased towards richness (Shannon), evenness or dominance (Simpson), or lose information by reducing the data through clustering to obtain OTU abundances for determining singletons and doubletons (Chao 1) [32]. Because abundant OTUs dominate the IKF, the *I*_kf_ is not very sensitive to the presence of rare OTUs or the ‘long tails’ frequently observed in microbial communities. Thus, while it is intuitively satisfying to capture multiple sources of diversity in a single value, the complex calculation of *I*_kf_ makes it difficult to attribute differences to a single source, such as OTU richness or phylogenetic diversity.

As a result, the value of the *I*_kf_ is difficult to assess without examining more applications. In the cases examined here, *K*-shuff analyses appeared to provide useful insights into the diversity of the microbial communities of cropland, pasture and forest soils as well as household surfaces. Moreover, it allowed comparisons of very different communities from seawater, marsh sediments and soils. In the long run, because of its high sensitivity and novel approach to structural diversity, it may provide new insights into the nature and structure of microbial communities. Although tested with 16S rRNA gene libraries, the methodology could be easily extended to other genes of interest and should be useful for other ecological and microbiological applications.

### Applicability of *K*-shuff

Compared to some methods, *K*-shuff is computationally expensive. However, the run times can be greatly reduced by subsampling large data sets. In our analysis of the subsampled dataset comprising 17366 sequences from Dunn et al. [23], the *I*_kf_ and *C*_kf_ were calculated in less than five minutes. However, calculating the p-values took about 4.5 h for the same dataset with 1000 permutations. With new programming languages and algorithms being developed, we believe that this limitation will be overcome soon.

As a test of the applicability of *K*-shuff, bacterial communities collected from a variety of soil types, land-use, vegetation, seasons and climates across the United States were analyzed. Although *K*-shuff detected significant differences between most of these communities, many of these differences were very small. Of particular interest was the similarity of many soil communities regardless of the differences in soil type, land uses, and regional climate. The soils with communities very different from this central tendency were of two types. The first included soils without a history of disturbance, such as native prairie in Kansas and the forest in Georgia. The second were soils with specific land management practices, such as the conifer plantation in Michigan or tillage with inorganic fertilizers in Georgia. The ability of *K*-shuff to clearly visualize distinctions between these communities will help describe the role of bacterial communities in soil fertility, crop productivity, sustainability, and other properties of great practical importance.

In conclusion, the power and sensitivity of *K*-shuff allows the comparison of both structure and composition of microbial communities from gene sequence libraries derived either by high-throughput methods (pyrosequencing and Illumina^®^) or Sanger methodology. *K*-shuff offers an ecologically meaningful ordination of structural and compositional differences through the use of multidimensional scaling of its two functions, the intra *K*-function (IKF) and cross *K*-function (CKF). Monte Carlo tests of the IKF and CKF can also be used to test for statistically significant differences. *K*-shuff was also found to be more sensitive than LIBSHUFF and UniFrac in detecting compositional differences. At the same time, its sensitivity allows an in-depth analysis of communities up to and within an OTU, which at times comprise multiple species, and dissection of small differences that might otherwise remain undetected. This is especially useful when comparing communities that are compositionally similar but functionally different. *K*-shuff will therefore prove beneficial for conventional microbiome analysis as well as specific hypothesis testing.

## Supporting Information

S1 File Supporting tables (A to D) and figures (A to O) for the main text.
(DOCX)
