# Supplementary material for: K-shuff: A Novel Algorithm for Characterizing Structural and Compositional Diversity in Gene Libraries
Source: PLoS One. 2016 Dec 2;11(12):e0167634. doi: 10.1371/journal.pone.0167634 (PMC5135132; doi:10.1371/journal.pone.0167634)
Supplement: S1 File — Supporting tables (A to D) and figures (A to O) for the main text. (DOCX) [file pone.0167634.s001.docx]

**SUPPLEMENTARY INFORMATION**

***K*-shuff: a novel algorithm for characterizing structural and compositional diversity in gene libraries**

1. **Power analysis and performance evaluation of *K*-shuff for clone libraries**

To evaluate effect of library size on the ability of K-shuff to detect differences in libraries, test 16S rRNA gene libraries prepared by Sanger sequencing were compiled by combining 1000 sequences from salt marsh sediments [18], forest and cropland soils [20], and seawater (unpublished data). Representative IKF and CKF plots of the complete forest and sediment libraries are shown below (Fig A). Power analysis was then performed to test the statistical power of K-shuff by comparing the soil and sediment or seawater and sediment communities. In this procedure, N (= 50, 75 and 100) sequences were randomly selected from the soil or sediment libraries (or seawater and sediment libraries) and mixed in different proportions (ω) to create pairs of libraries. One library in each pair consists of ωN soil sequences and (1-ω)N sediment sequences, while the other library in the pair contains (1-ω)N soil sequences and ωN sediment sequences. Then ω was increased from 0.5 to 1 in steps of 0.05, resulting in pairs of libraries with various levels of dissimilarity. With ω=0.5, the two generated libraries should be similar in both structure and membership, and the libraries should be very different when ω=1; i.e. no mixing between communities. For each combination of N and ω, the $T_{I}$ and $T_{c}$ test statistics were performed on 1000 pairs of mixed libraries and the power calculated.


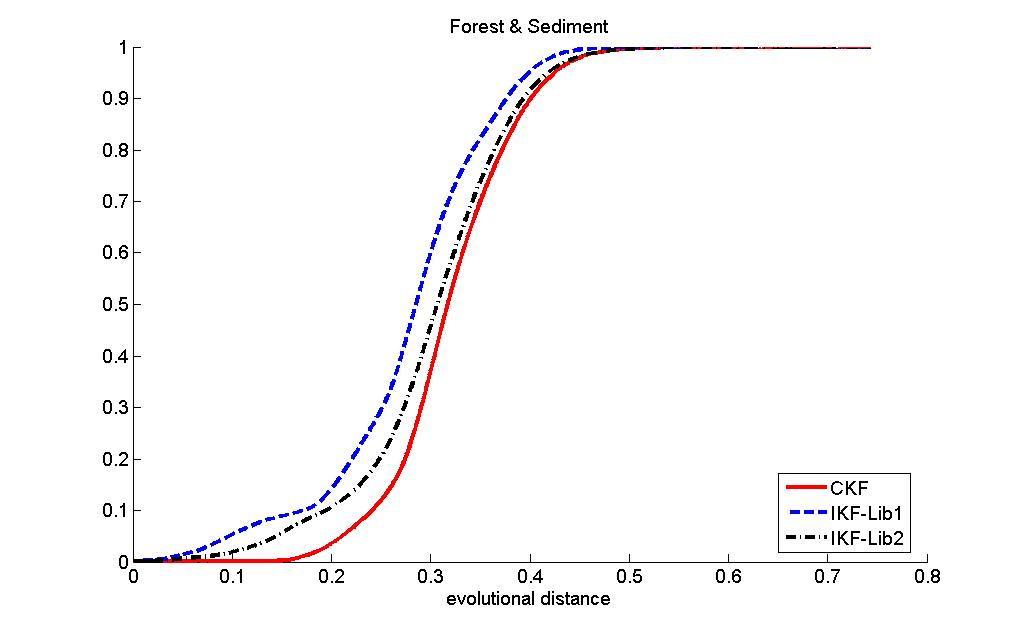


**Figure A.** IKFs and CKF for the soil (library 1) and the sediment (library 2) libraries. The IKFs and CKF were calculated for with entire 1000 sequences in each library. The structure (IKF) and compositions (CKF) of the libraries were significantly different, both with p-values of <0.001

**Table A.** Power analysis of seawater vs. salt marsh sediment libraries: Power (# of times rejecting H0/1000), p < 0.05, N=number of sequences in each library.

| Proportion (ω) | IKF | | | CKF | | |
| --- | --- | --- | --- | --- | --- | --- |
|  | N=50 | N=75 | N=100 | N=50 | N=75 | N=100 |
| 50% | 0.012 | 0.017 | 0.012 | 0.046 | 0.036 | 0.042 |
| 55% | 0.028 | 0.047 | 0.078 | 0.034 | 0.048 | 0.053 |
| 60% | 0.193 | 0.299 | 0.428 | 0.093 | 0.211 | 0.406 |
| 65% | 0.408 | 0.655 | 0.865 | 0.390 | 0.724 | 0.950 |
| 70% | 0.782 | 0.945 | 0.994 | 0.923 | 0.986 | 0.999 |
| 75% | 0.944 | 0.997 | 0.999 | 0.988 | 1.000 | 1.000 |
| 80% | 0.996 | 1.000 | 1.000 | 1.000 | 1.000 | 1.000 |
| 85% | 0.999 | 1.000 | 1.000 | 1.000 | 1.000 | 1.000 |
| 90% | 1.000 | 1000 | 1.000 | 1.000 | 1000 | 1.000 |
| 95% | 1.000 | 1.000 | 1.000 | 1.000 | 1.000 | 1.000 |
| 100% | 1.000 | 1.000 | 1.000 | 1.000 | 1.000 | 1.000 |

**Table B.** Power analysis of soil vs. salt marsh sediment libraries: Power (# of times rejecting H0/1000), p < 0.05, N=number of sequences in each library.

| Proportion (ω) | IKF | | | CKF | | |
| --- | --- | --- | --- | --- | --- | --- |
|  | N=50 | N=75 | N=100 | N=50 | N=75 | N=100 |
| 50% | 0.046 | 0.044 | 0.045 | 0.019 | 0.033 | 0.030 |
| 55% | 0.051 | 0.044 | 0.045 | 0.022 | 0.049 | 0.053 |
| 60% | 0.066 | 0.050 | 0.051 | 0.106 | 0.184 | 0.255 |
| 65% | 0.078 | 0.059 | 0.078 | 0.251 | 0.452 | 0.800 |
| 70% | 0.102 | 0.080 | 0.114 | 0.698 | 0.901 | 0.998 |
| 75% | 0.107 | 0.117 | 0.150 | 0.925 | 1.000 | 1.000 |
| 80% | 0.144 | 0.144 | 0.215 | 0.995 | 1.000 | 1.000 |
| 85% | 0.162 | 0.206 | 0.256 | 1.000 | 1.000 | 1.000 |
| 90% | 0.203 | 0.277 | 0.348 | 1.000 | 1.000 | 1.000 |
| 95% | 0.231 | 0.341 | 0.433 | 1.000 | 1.000 | 1.000 |
| 100% | 0.313 | 0.409 | 0.533 | 1.000 | 1.000 | 1.000 |

**
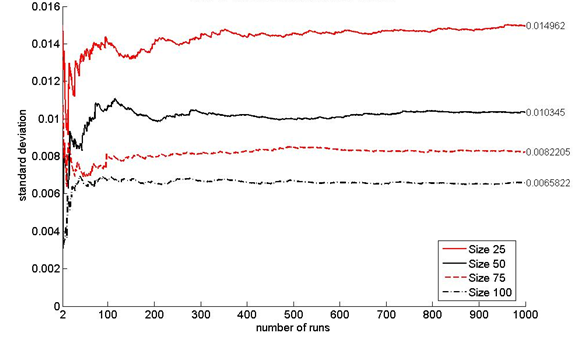
**

**
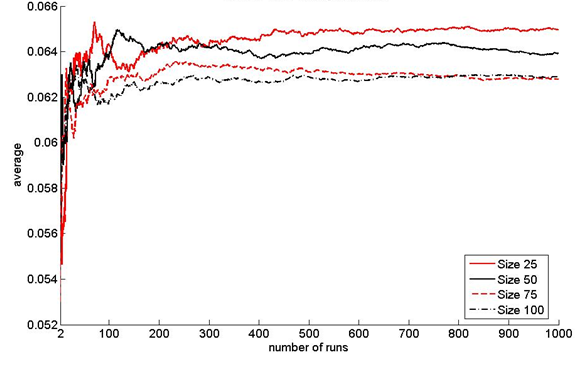
**

**Figure B.** Reproducibility and effect of sample size on the *C*_kf_ comparison of soil and marsh sediment libraries. The estimators were calculated for 1000 randomly chosen subsamples of the soil and marsh sediment rRNA gene libraries. For each simulation, a total of N sequences are randomly drawn from each of the two libraries (soil and sediment); N = 25, 50, 75, and 100. For the two libraries of N sequences, the *C*_kf_ was calculated. The cumulative average and standard deviations for the first *i* simulations were then calculated and plotted; *i*=2, 3, …, 1000.

**
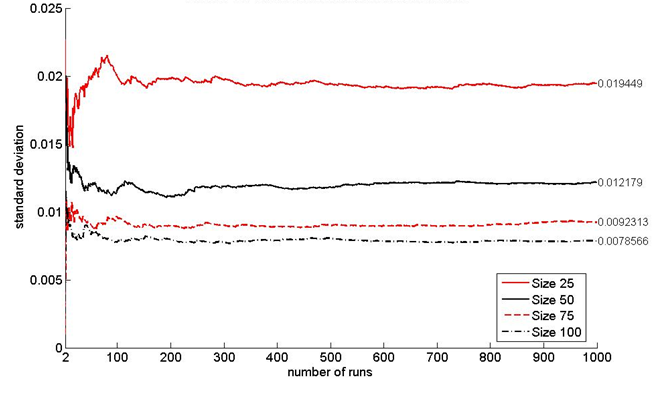

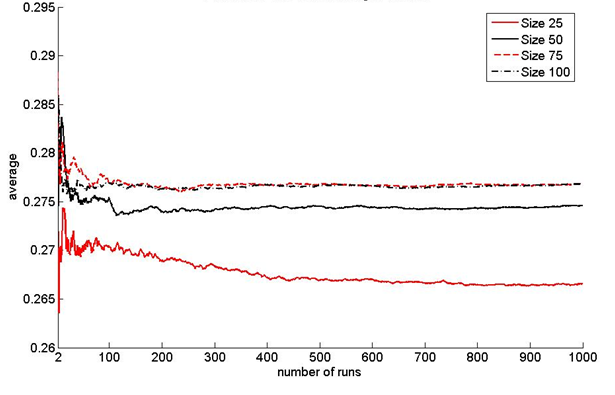
**

**Figure C.** Reproducibility and effect of sample size on the *I*_kf_ comparison of the soil libraries. Other details in the calculation as in Fig B.


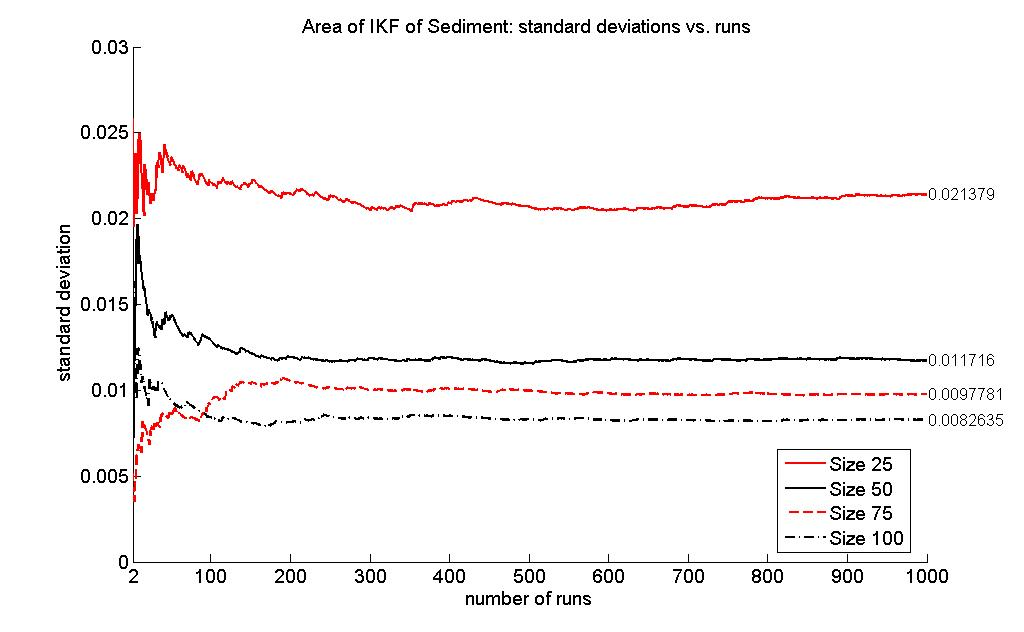


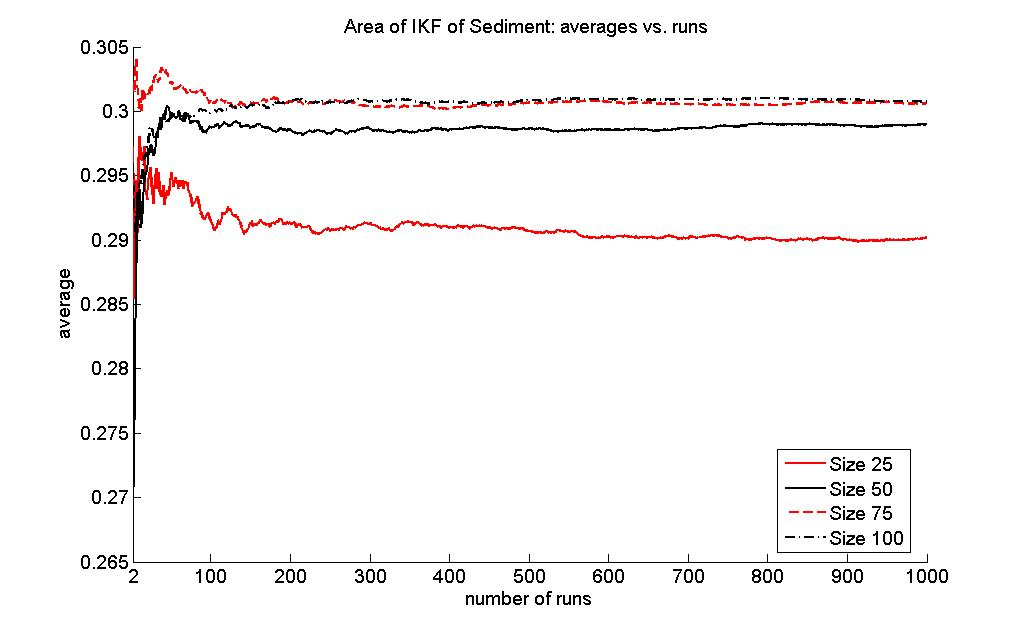


**Figure D.** Reproducibility and effect of sample size on the *I*_kf_ comparison of marsh sediment libraries. Other details in the calculation as in Fig B.


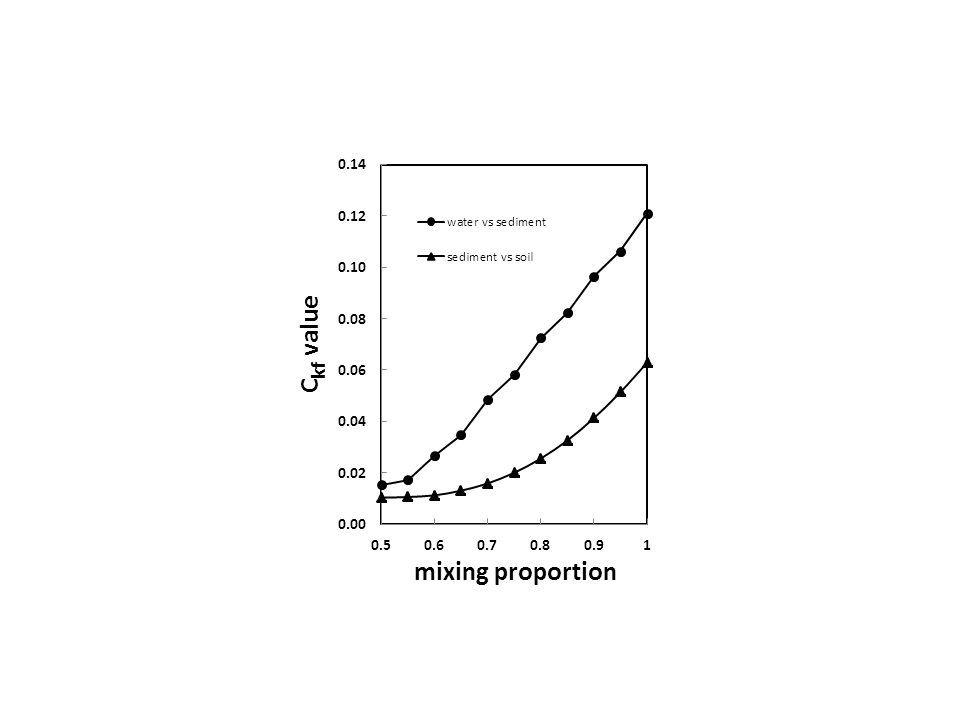


**Figure E.** The relationship between *C*_kf_ and the mixing proportions (ω) for seawater, sediment, and soil libraries. The *C*_kf_ was calculated during the power analyses in Tables A and B for N = 100.


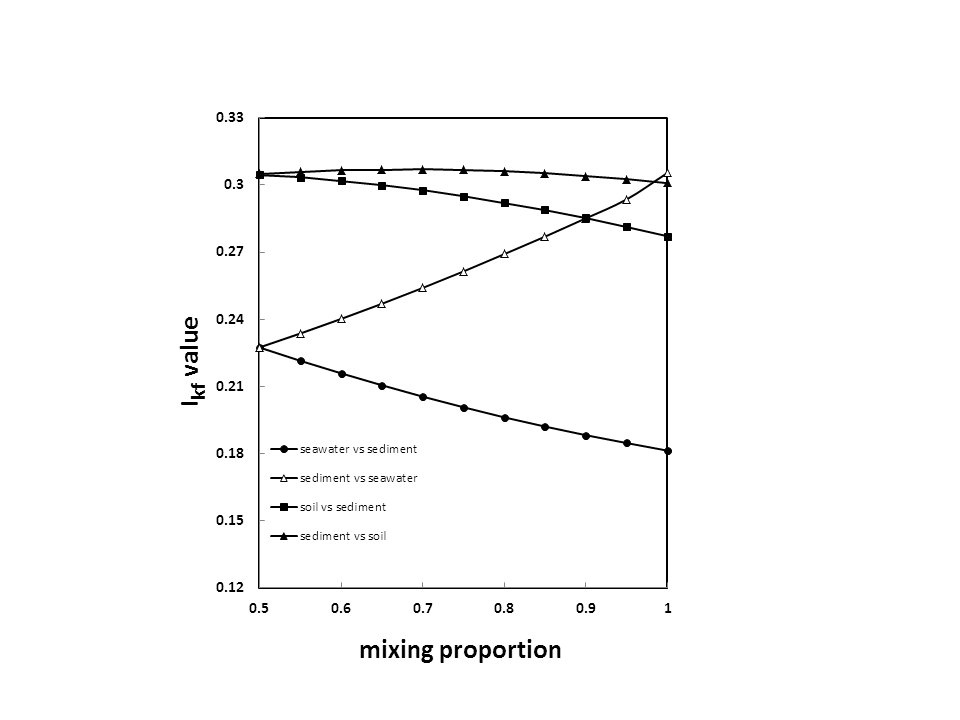


**Figure F.** The relationship between *I*_kf_ and the mixing proportions (ω) for seawater, sediment, and soil libraries. The *I*_kf_ was calculated during the power analyses in Tables A and B for N = 100.

1. **Evaluation of *K*-shuff for Illumina sequencing and power analyses**

**Figure G.** Effect of sample size on the mean *I*_kf_ values across the subsampled Illumina dataset. The effect of the number of sequences selected to calculate the *I*_kf_ was determined from random samples of 100, 200, 300, 400 and 500 sequences for Cutting board (19 samples), Door Exterior (18 samples) and Pillowcase (20 samples) communities. These sites were chosen because they possessed low, high and intermediate OTU richness, respectively (see Fig 1 in Dunn et al. [23]). Bars represent the minimum and maximum values. *Excludes lowest outliers in accordance with Fig 1 in Dunn et al. [23].

**Figure H.** Reproducibility and effect of sample size on the *C*_kf_ comparison of Cutting board (Cb) and Pillowcase (Pc) libraries. The estimators were calculated for 1000 randomly chosen subsamples of the Cb and Pc gene libraries. For each simulation, a total of N sequences are randomly drawn from each of the two libraries (Cb and Pc); N = 50, 75, and 100. For the two libraries of N sequences, the *C*_kf_ was calculated. The cumulative average and standard deviations for the first *i* simulations were then calculated and plotted; *i*=2, 3, …, 1000.

**Figure I.** Reproducibility and effect of sample size on the *I*_kf_ comparison of the Cutting board libraries. For other details, see the legend to Fig H.

**Figure J.** Reproducibility and effect of sample size on the *I*_kf_ comparison of Pillowcase libraries. For other details, see the legend to Fig H.

**Power analysis.** For the Illumina dataset, the statistical power of K-shuff was tested by comparing the cutting board (Cb) and pillowcase (Pc) libraries at different mixing proportions (ω= 0.5 to 1) and sizes (N= 50, 75, 100). A Perl script was written to run 1000 iterations for each combination of ω and N. For each iteration, the script randomly sampled the desired number of sequences from the two libraries and build a squared distance matrix using MOTHUR v.1.35.1 run in batch mode. It then ran *K*-shuff, and extracted the test statistics (*T_I_* and *T_C_*) and *p*- values.

**Table C.** Power analysis of Cutting board vs. Pillowcase libraries: Power (# of times rejecting H0/1000), p < 0.05, N=number of sequences in each library.

|  | IKF | | | CKF | | |
| --- | --- | --- | --- | --- | --- | --- |
| Proportion (ω) | N=50 | N=75 | N=100 | N=50 | N=75 | N=100 |
| 50% | 0.039 | 0.037 | 0.042 | 0.044 | 0.028 | 0.057 |
| 55% | 0.044 | 0.048 | 0.042 | 0.043 | 0.042 | 0.030 |
| 60% | 0.057 | 0.073 | 0.082 | 0.067 | 0.057 | 0.056 |
| 65% | 0.095 | 0.130 | 0.146 | 0.070 | 0.077 | 0.108 |
| 70% | 0.126 | 0.177 | 0.214 | 0.084 | 0.128 | 0.151 |
| 75% | 0.175 | 0.242 | 0.336 | 0.132 | 0.211 | 0.280 |
| 80% | 0.246 | 0.354 | 0.473 | 0.194 | 0.336 | 0.421 |
| 85% | 0.308 | 0.443 | 0.609 | 0.281 | 0.433 | 0.667 |
| 90% | 0.425 | 0.597 | 0.732 | 0.416 | 0.645 | 0.873 |
| 95% | 0.473 | 0.699 | 0.860 | 0.525 | 0.826 | 0.966 |
| 100% | 0.607 | 0.812 | 0.920 | 0.705 | 0.935 | 0.997 |

**
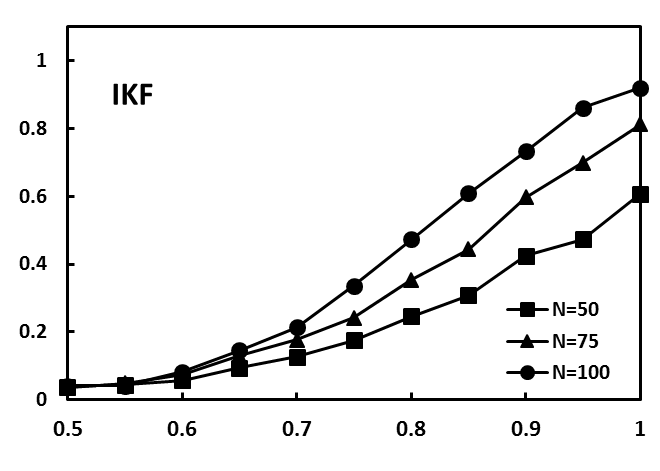
**

**Fraction significantly different**

**Mixing proportion**

**Figure K.** Power of *T_I_* (IKF) and *T_C_* (CKF) to distinguish libraries of bacterial 16S rRNA gene sequences from cutting board (Cb) and pillowcase (Pc) communities. The fraction of 1000 K-shuff comparisons that were significantly different or the power compared to the mixing proportion (ω) of the libraries as in Table C.

**Figure L.** The relationship between *C*_kf_ and the mixing proportions (ω) for Cutting board and Pillowcase libraries. The *C*_kf_ was calculated during the power analyses in Table C.

**Figure M.** The relationship between *I*_kf_ and the mixing proportions (ω) for Cutting board (Cb) and Pillowcase (Pc) libraries. The *I*_kf_ was calculated during the power analyses in Table C.

1. **Application of *K-*shuff to Illumina datasets**


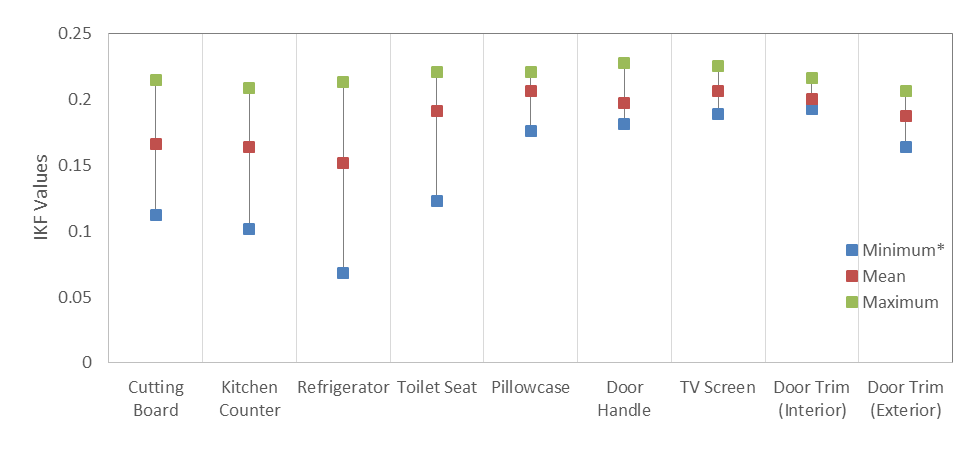


**Figure N.** *I*_kf_ values for each of the nine household sites examined by Dunn et al. [23]. The *I*_kf_ values were calculated for 100 sequences from each of the 40 households at each site. The mean, minimum and maximum values are shown. *Excludes lowest outliers in accordance with Fig 1 in Dunn et al. [23].

**Figure O.** MDS plot of the household bacterial communities with the compositional parameter *C*_kf_ for the subsampled Illumina^®^ libraries by Dunn et al. [23]. The *C*_kf_ values were calculated for all 174 samples each with 100 sequences.

**Table D.** *C*_kf_ (bottom left) and corresponding p-values (upper right) for OTU1_0.03_ at each site. The number of sequences at each site is in parenthesis in the first column.

|  | **Cb** | **Dh** | **Di** | **Do** | **Fr** | **Kc** | **Pc** | **Ts** | **Tv** |
| --- | --- | --- | --- | --- | --- | --- | --- | --- | --- |
| **Cb (53)** |  | 0.0310 | 0.0280 | 0.3040 | 0.0060 | 0.0140 | 0.1500 | 0.5990 | 0.3260 |
| **Dh (63)** | 0.0015 |  | 0.0890 | 0.2250 | 0.0010 | 0.0080 | 0.3300 | 0.2180 | 0.4350 |
| **Di (17)** | 0.0053 | 0.0038 |  | 0.2360 | 0.0010 | 0.0020 | 0.7880 | 0.0030 | 0.3630 |
| **Do (1)** | 0.0527 | 0.0529 | 0.0520 |  | 1.0000 | 0.9690 | 0.2070 | 0.6750 | 0.3730 |
| **Fr (417)** | 0.0048 | 0.0052 | 0.0085 | 0.0533 |  | 0.6450 | 0.0010 | 0.5660 | 0.0070 |
| **Kc (192)** | 0.0043 | 0.0046 | 0.0079 | 0.0533 | 0.0005 |  | 0.0010 | 0.8400 | 0.0190 |
| **Pc (74)** | 0.0035 | 0.0028 | 0.0018 | 0.0522 | 0.0080 | 0.0074 |  | 0.1020 | 0.8650 |
| **Ts (40)** | 0.0035 | 0.0038 | 0.0072 | 0.0532 | 0.0013 | 0.0008 | 0.0066 |  | 0.0630 |
| **Tv (40)** | 0.0022 | 0.0012 | 0.0037 | 0.0527 | 0.0048 | 0.0043 | 0.0032 | 0.0035 |  |
